# Chinese Herbal Decoction Based on Syndrome Differentiation as Maintenance Therapy in Patients with Extensive-Stage Small-Cell Lung Cancer: An Exploratory and Small Prospective Cohort Study

**DOI:** 10.1155/2015/601067

**Published:** 2015-03-01

**Authors:** Rui Liu, Shu lin He, Yuan chen Zhao, Hong gang Zheng, Cong huang Li, Yan ju Bao, Ying gang Qin, Wei Hou, Bao Jin Hua

**Affiliations:** ^1^Oncology Department of Guang'anmen Hospital, China Academy of Chinese Medical Sciences, Beijing 100053, China; ^2^Cancer Institute, China Academy of Chinese Medical Sciences, Beijing 100053, China; ^3^Beijing University of Chinese Medicine, Beijing 100029, China

## Abstract

*Objective*. To investigate the treatment effect and treatment length of Chinese herbal decoction (CHD) as maintenance therapy on patients with extensive-stage small-cell lung cancer (ES-SCLC) and to reflect the real syndrome differentiation (Bian Zheng) practices of traditional Chinese medicine (TCM). *Patients and Methods*. Different CHDs were prescribed for each patient based on syndrome differentiation. The length of CHD treatment was divided into two phases for analyzing progression-free survival (PFS) and postprogression survival (PPS). *Results*. Three hundred and fifty-seven CHDs were prescribed based on syndrome differentiation during the study period. Median PFS was significantly longer in patients who received CHD >3 months than patients who received CHD ≤3 months in the first phase (8.7 months versus 4.5 months; hazard ratio (HR), 0.52; 95% confidence interval (CI), 0.41–0.99; *P* = 0.0009). Median PPS was significantly longer in patients who received CHD >7 months than patients who received CHD ≤7 months in the second phase (11.7 months versus 5.1 months; HR, 2.32; 95% CI, 1.90–2.74; *P* = 0.002). *Conclusion*. CHD could improve PFS and PPS, which are closely related to treatment time and deepness of response of first-line therapy. In addition, CHD could improve body function and keep patients in a relatively stable state.

## 1. Introduction

Lung cancer is a leading cause of cancer-related death in the world [1, 2]. Small-cell lung cancer (SCLC) represents approximately 15% of all lung cancer cases [3]. Between 60% and 70% of these patients have extensive disease at diagnosis, with metastases that involve one or more sites, such as brain, liver, bone, or bone marrow [4]. The clinical outcome of extensive-stage small-cell lung cancer (ES-SCLC) is poor, with a median survival time of only 6–8 weeks without treatment [5]. The combination chemotherapy of platinum and etoposide is still the first-line therapy, with a median overall survival (OS) of 7–11 months and 2-year survival rates of 1–5% [6, 7]. Although SCLC is a chemosensitive malignancy with an overall response rate of 60–80% in patients with ES-SCLC [5], the response duration in most patients is usually short, with a progression-free survival (PFS) of about 4–6 months [8, 9]. About 80% of limited-stage small-cell lung cancer (LS-SCLC) and almost all the ES-SCLC patients have a relapse or progression after treatment in 1 year, and approximately 95% of them eventually die from disease progression [5].

Although new drugs have been developed and the remission rate is 94% [10], improvements in ES-SCLC patients OS are still extremely limited. Clinical practitioners were not successful with EP Plan plus topotecan, paclitaxel, irinotecan, or anti-Bcl-2 [11–14]. To date, no clear benefits to OS with maintenance chemotherapy have been confirmed. More than 10 clinical trials studied the efficacy of the SCLC patients (including the LS-SCLC and ES-SCLC) who continued maintenance chemotherapy after conventional chemotherapy treatment of four to six cycles. Most results showed that maintenance chemotherapy has little benefit in improving survival rates but increases side effects [15–17]. The chemotherapy of SCLC has its limitations, so extending patient lifespan by using treatments with lower toxicities is necessary. This may be of significant clinical value in prolonging patient survival time (including PFS and postprogression survival, (PPS)).

Chinese herbal medicines (CHM) have been used for thousands of years and play an indispensable part of alternative medicine and a vital role in adjuvant therapy of tumors [18]. CHM in conjunction with chemotherapy demonstrated significant improvements in quality of life and a reduction in anemia and neutropenia [19]. Because of its low cost and toxicity, abundance, and effectiveness [20, 21], CHM is often used in cancer patients who have finished conventional treatment, such as surgery, chemotherapy, or radiotherapy and acts as maintenance treatment. Recent clinical observation provided several cases of CHM prolonging ES-SCLC patients' OS and raising survival rate. For example, compared with chemoradiotherapy alone and CHM followed by chemoradiotherapy, the median overall survival was 7.6 months and 11.1 months and 1-year survival rate was 18.2% and 38.6% [22]. Other studies have shown that CHM combined with chemotherapy could reduce toxic effects and improve quality of life. However, because of poor quality trials and low sample sizes, higher quality randomized, double-blind, controlled clinical trials are required to get more comprehensive and objective conclusions [23]. The ingredients of Chinese patent medicines (CPM) are relatively fixed and easily applied, and some trials focusing on CPM have been conducted, including shen-qi-fu-zheng injection, compound* Radix Sophorae flavescentis* injection, and ai-kang injection [23]. However, studies have not objectively explored the efficacy of CHM treating ES-SCLC. In China, CHM is the main form of traditional Chinese clinical treatment (including the treatment of tumors), and it is widely used in clinical practice.

CHM treatments should be tailored to fit the individual clinical presentations of patients, even though they all may have the same medical diagnosis [18, 24]. According to the fundamental principles of traditional Chinese medicine, CHM should be based on “syndrome differentiation.” A TCM syndrome (Zheng) is the abstraction of a major disharmonious pathogenesis and is an outcome after analyzing all symptoms and signs. All therapeutic methods in TCM come from syndrome differentiation, and it is based on each patient's different symptoms, tongue coating, and pulse condition. When herbs are called for, several are usually used together, and the whole herbs are used, not purified compounds [20]. This type of CHM may also be called Chinese herbal decoction (CHD). CHD is the main TCM treatment method based on syndrome differentiation, according to the assessment of the pattern of symptoms manifested in each individual. CHD has an advantage in that doctors can add or decrease the type and amount of Chinese herbs in addition to the fundamental formula to make the decoction more suitable for each patient's condition.

The treatment length of CHD is also a factor for each patient. At present, the Chinese Tumor Research Academy of the Traditional Chinese Medicine Institute suggests that the treatment length of CHD is set by the point of tumor recurrence. That is, twice a day in the first 1–3 years, once a day in next 3–5 years, and then no CHD after 5 years. However, this is an expert consensus, without evidence-based data. We found in our clinical practice that the SCLC patients who take CHD as sustaining treatment after chemoradiotherapy substantially prolonged OS. Therefore, we organized a small prospective cohort to systemically evaluate the value of CHD and its treatment time on ES-SCLC.

We aimed to explore the effect of CHD as maintenance therapy on PFS and PPS in ES-SCLC patients with first-line chemotherapy response. The changes in TCM syndromes and PS during CHD treatment were also observed, including the function of CHD in reducing the toxic effects of chemotherapy and its safety. This study gives evidence for the clinical daily practice of TCM in the treatment of tumors and uncovers the efficacy of the fundamental formula to provide further evidence for basic research and clinical practice in the future.

## 2. Patients and Methods

### 2.1. Study Protocol

This study was an exploratory, prospective, and small cohort clinical observation, using traditional Chinese medicine (TCM) as maintenance therapy in comprehensive treatment of ES-SCLC. Patients were evaluated for response (according to RECIST 1.1 (Response Evaluation Criteria in Solid Tumors)) after the completion of two chemotherapeutic courses. Twenty-eight eligible patients who underwent TCM treatment from January 2010 through March 2012 at our clinic, Oncology Department of Guang'anmen Hospital, China Academy of Chinese Medical Sciences (GAMH, CACMS), were enrolled in this study. The comprehensive treatment team included Guang'anmen Hospital, China Academy of Chinese Medical Sciences, Cancer Hospital, Chinese Academy of Medical Sciences, Beijing Cancer Hospital, and other local cancer hospitals, and involved TCM doctors, chemotherapy physicians, and radiotherapy doctors.

**Figure 1:**
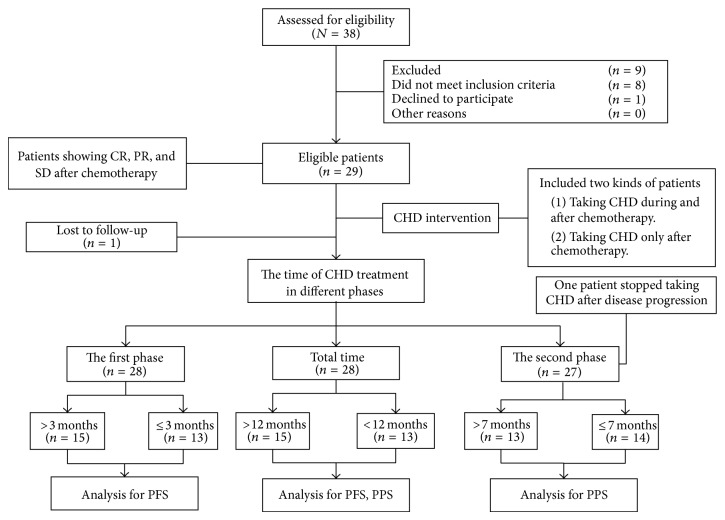
The study was separated into two phases. The first phase was from the beginning of CHD treatment to disease progression. The second phase was from disease progression to death or study conclusion (September, 2013). Total time was the time of CHD treatment in the first phases and second phases. The length of CHD treatment was grouped according to the median CHD treatment time in different phases.

This study included two types of patients: those taking CHD during and after chemotherapy and those who took CHD only after chemotherapy. The length of CHD treatment and follow-up was separated into two phases to analyze the survival time in each phase. The first phase was from the beginning of CHD treatment to disease progression. The second phase was from disease progression to death or study conclusion (September, 2013). After disease progression, we would inform patients and recommend CHD treatment. One patient stopped taking CHD after disease progression. Patients were suggested to take the decoction for at least 1 year, but patients ultimately decided the length of their own CHD treatment. All patients were given informed consent before enrollment (Figure 1).

### 2.2. Patients

To be eligible for the study, patients had to fulfill the following inclusion criteria. (1) They should have a histologically or cytologically confirmed diagnosis of ES-SCLC according to Veterans Administration Lung Study Group staging criteria. This included patients with pleural or pericardial effusions and/or supraclavicular lymphadenopathy. (2) They should have an effective response after first-line chemotherapy, including complete response (CR), partial response (PR), or stable disease (SD); (3) have a combination of platinum and etoposide as first-line chemotherapy; (4) have a life expectancy of at least 3 months; (5) have the ability to swallow and retain oral medication; (6) have a World Health Organization performance status (PS) of 0–2, aged >18 years and <80 years; and (7) have adequate hematologic, hepatic, and renal function and coagulation parameters.

Patients were excluded if they met any of the following: if they had an ineffective response of progressive disease (PD) after two cycles of first-line chemotherapy; had a history of taking TCM or other treatments after diagnosis; had known hypersensitivity to Chinese herbs (CH); had a history of recent myocardial infarction, congestive heart failure, or arrhythmia that required medical treatment; had an active infection; had any malignancy other than SCLC; if they were unable to comply with the treatment; if the disease progressed under CHD treatment. Children, pregnant, or lactating women and psychiatric patients were also excluded from the study. Withdrawal from the trial was considered if patients demonstrated significant noncompliance with the protocol requirement or experienced unacceptable toxicities or adverse events (AEs).

### 2.3. Herbal Treatment

The comprehensive treatment plan is composed of several therapies, including TCM, chemotherapy, and radiotherapy. The chemoradiotherapy plan was made and carried out by the professional doctors from the China Academy of Chinese Medical Sciences, Cancer Hospital, Chinese Academy of Medical Sciences, Beijing Cancer Hospital, and other local cancer hospitals.

We made and adjusted the TCM prescriptions based on each patient's syndrome differentiation. The basic syndrome pattern of SCLC includes Qi deficiency, Yin deficiency, and Phlegm syndrome. Qi deficiency manifests as the following: cough, shortness of breath, fatigue and weakness, spontaneous sweating, pale tongue, thin coating, and a weak pulse. Yin deficiency manifests as the following: characteristically dry cough with little phlegm, dry mouth, red tongue, less tongue coating, and a thready and rapid pulse. Phlegm syndrome manifests as the following: cough, constant phlegm, white and greasy coating, and a slippery pulse. Based on the fundamental prescriptions (Table 1), the prescription might be modified as follows.

For dusky tongue use* Curcuma zedoaria* (E Zhu) 12 g and shelled walnut (Tao Ren) 10 g. For cough add Folium Eriobotryae (Pi Pa Ye) 12 g, Thunberg Fritillary Bulb (Zhe Bei Mu) 30 g, Michaelmas daisy (Zi Yuan) 15 g, and Flos Farfarae (Kuan Dong Hua) 15 g. For hemoptysis add Lalang grass rhizome (Bai Mao Gen) 30 g and notoginseng powder (San Qi Fen) 9 g. For chest pain add* Rhizoma Corydalis* (Yuan Hu) 20 g and* Radix Clematidis* (Wei Ling Xian) 15 g. For pleural effusion add* Semen Lepidii* (Ting Li Zi) 15 g,* Radix Stephaniae Tetrandrae* (Fen Fang Ji) 15 g,* Zanthoxylum bungeanum* Maxim (Jiao Mu) 9 g,* Rhizoma Alismatis* (Ze Xie) 15 g, and* Herba Lycopi* (Ze Lan) 12 g. For brain metastases add* Gastrodia elata* (Tian Ma) 15 g,* Uncaria rhynchophylla* (Gou Teng) 15 g,* Scorpio* (Quan Xie) 3 g, and* Lumbricus* (Di Long) 3 g. For patients with a fever add* Radix Scutellariae* (Yin Chai Hu) 15 chd15 g,* Artemisia apiaceae (Qin Hao) 30 g, and Rhizoma* Anemarrhenae (Zhi Mu) 12 g. For poor diet, add fried rice sprout (Chao Gu Ya) 15 g, colored malt (Chao Mai Ya) 15 g, scorched hawthorn fruit (Jiao Shan Zha) 15 g, and medicated leaven (Jiao Shen Qu) 15 g. For poor sleep, add spine date seed (Suan Zao Ren) 30 g,* Concha Margaritifera Usta* (Zhen Zhu Mu) 30 g, and* Polygala tenuifolia* (Zhi Yuan Zhi) 9 g. For constipation, add rhubarb root parched in wine (Jiu Da Huang) 12 g, and* Semen Cannabis* (Huo Ma Ren) 30 g. Each prescription had one to two types of anticancer CH (Table 1(d)). All Chinese herbal medicines were supplied by the pharmacy of GAMH, CACMS.

### 2.4. Efficacy Assessments

The primary endpoint was PFS. PFS is defined as the time from the beginning of CHD treatment until disease progression or death from any cause and confirmed on the last CT before progressive disease. The second primary endpoint was PPS, defined as the time from disease progression to death or study conclusion (September, 2013). Secondary endpoints included the following: overall survival (OS), defined as the time from the date of first-line treatment until death or the study deadline; 24-week PFS rate; one-year survival rate; and functional assessment, which included PS and TCM syndromes.

TCM syndromes were measured by fatigue, cough, shortness of breath, expectoration, panting, chest pain, loss of appetite, insomnia, and constipation. Degrees of TCM syndromes were divided into four ranks: asymptomatic (score = 0), mild (score = 1), moderate (score = 2), and serious (score = 3). The scores were recorded before and after treatment. A clinical symptom score that decreased by 2/3 or more indicates that the symptoms were significantly alleviated. A score decrease by more than 1/3 but less than 2/3 indicates partial alleviation of symptoms. A score decrease of less than 1/3 indicates no relief. The number of alleviated cases equals the number of significantly alleviated cases added to the number of partially alleviated cases.

### 2.5. Safety Evaluations

Adverse events (AE) were observed as the second endpoint. Safety assessments were performed on an ongoing basis throughout the trial. During chemotherapy, AEs were evaluated using the National Cancer Institute Common Terminology Criteria for Adverse Events, version 3.0.28. Liver and renal function and blood routine tests were conducted every 3 months while taking CHD to look for chronic cumulative toxicity.

### 2.6. Follow-Up

The first phase of follow-up was from the beginning of CHD treatment to disease progression. The second phase was from disease progression to patient death or study conclusion. The follow-up was conducted as an outpatient service and telephone interview to record any changes in the patient's symptom scores and PS scores. Follow-up evaluation included a complete medical history and physical examination, blood chemistry tests (including complete blood cell counts, tumor markers, liver, and renal function tests), chest CT scan, and abdominal CT scan or ultrasonography at 6 weeks. If no progression had occurred after 6 months of follow-up, then imaging was performed every 12–16 weeks until progression according to National Comprehensive Cancer Network (NCCN) Guidelines. A more sophisticated workup was performed only if indicated.

### 2.7. Statistical Analysis

All efficacy analyses were performed in the intent-to-treat (ITT) population, which was composed of all enrolled patients who had a baseline measurement and at least one measurement while being on the study drug. The study is not designed to provide anything other than very preliminary exploratory data on feasibility and as a reflection of clinical practice including differential diagnosis, herb selection, and exposure duration. Descriptive statistics have been used to analyze the baseline characteristics of the participants. Survival rates were estimated using the Kaplan-Meier method, and the log-rank test was used to compare survival curves. Survival curves were plotted with Prism 5.0 software (GraphPad, La Jolla, CA, USA). For normally distributed data, the independent Student's* t*-test was applied. All analyses were done using SPSS 18.0 (SPSS Inc., Chicago, IL, USA); all tests were two-sided and *P* values less than 0.05 were considered to be statistically significant.

## 3. Results

### 3.1. Baseline Characteristics of Study Groups

Between January 2010 and March 2012, 28 enrolled patients were eligible for analysis. The study included 22 men and 6 women, with a median age of 61 years (range, 42–75 years). The patients were in a good PS of 1-2. Eleven (39%) patients, 16 (57%) patients, and 1 (4%) patient were diagnosed by thoracocentesis, bronchoscopy, and phlegm, respectively. The median number of metastatic sites was two (range, 1–6). The most common sites of metastases included lymph nodes (100%) and then lung (54%). The median number of concomitant diseases was 1 (range, 0–4); 8 (29%) patients had diabetes mellitus, 4 (14%) had hypertension and other diseases including cardiac arrhythmia (4%), obliterative inflammation (4%), chronic bronchitis (4%), and emphysema (4%).

The study population was well balanced for performance status, first-line treatment, and efficacy evaluation (Table 2). All patients received first-line chemotherapy with four to six cycles of cisplatin or carboplatin and etoposide, and 22 patients were treated by second-line chemotherapy after disease progression with topotecan or other drugs. Twenty-five (89%) patients received cisplatin and etoposide, while the other 3 (11%) patients were treated with carboplatin and etoposide. Fifteen (54%) patients were administered with radiotherapy for a preventive palliative goal to prolong the patient's PFS and OS and to increase the rate of response. All patients were considered evaluable for response. Four patients (14%) achieved CR, 15 (54%) achieved PR, and 9 (32%) had SD.

### 3.2. Fundamental Prescription and Symptom Response

In the whole course of CHD treatment, we made diagnoses according to syndrome differentiations 357 times in total with an average of 12.8 per patient. Fundamental prescriptions of Qi deficiency, Yin deficiency, and Phlegm were used 187, 103, and 67 times, respectively. At first visit, the median number of symptoms was two (range 0–12). Other symptoms included nausea, acid reflux, spontaneous sweating, lumbar pain, upset, dry mouth, and bitter mouth. The major symptoms of these patients were fatigue (75%) and cough (64%), and 61% of the patients got shortness of breath, constipation, and poor appetite. Detailed information about the symptoms before and after CHD treatment and response rate are shown in Table 3.

### 3.3. Length of CHD Treatment

The median total length of CHD treatment was 12.2 months (range, 3.2–27 months). For the first phase (before disease progression), the median length of CHD treatment was 3.4 months. The group with >3 months had a median time of 6.7 months (Table 4). For the second phase (after disease progression), the median time of CHD treatment was 7.0 months. The group with >7 months had a median time of 12.7 months. Twenty-eight patients took CHD before the disease progression. One patient stopped taking CHD after disease progression.

### 3.4. Efficacy Results

The overall follow-up period was 8–27 months with a median of 14 months. All patients were alive in the first stage of follow-up with a median time of 3.4 months (range, 1–11.1 months). During the second stage of follow-up, 23 patients (82.1%) died. The cause of death in 21 patients was progression of disease, and the remaining two patients died from heart failure and infection.

**Figure 2:**
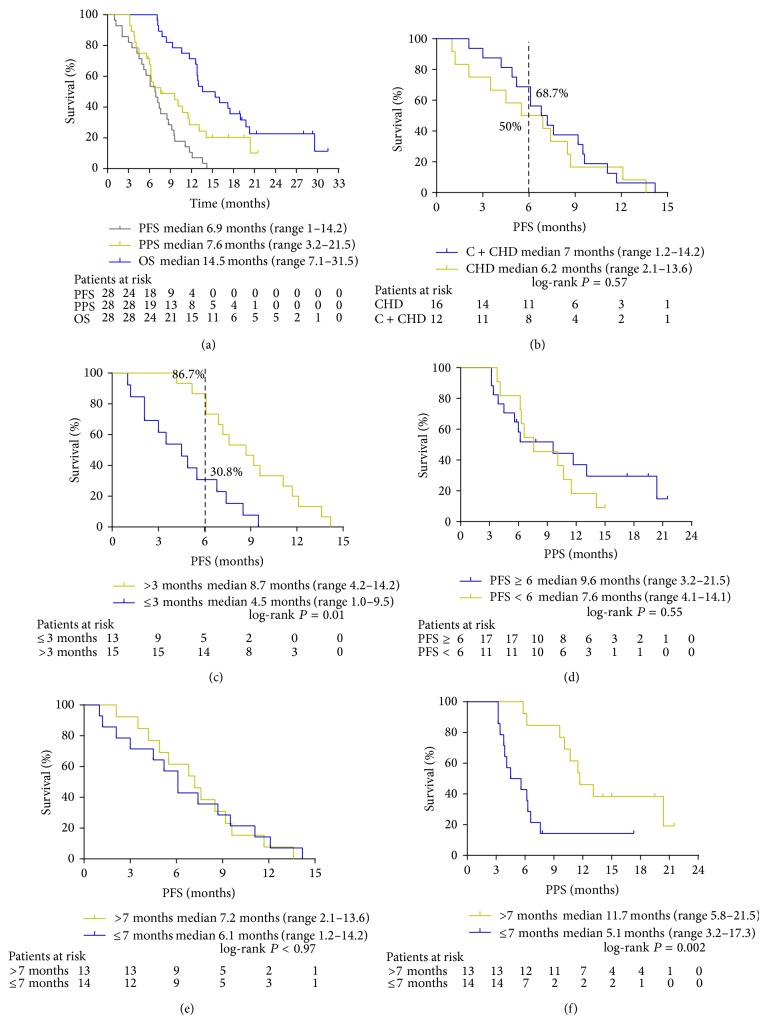
(a) Median PFS, PPS, and OS were 6.9 months (95% CI, 5.0–8.6 months), 7.6 months (95% CI, 5.5–9.7 months), and 14.5 months (95% CI, 10.6–18.6 months). (b) Patients were grouped by CHD during and after chemotherapy for PFS. (c) In the first phase (the time before disease progression), patients were grouped by CHD >3 months and CHD ≤3 months for PFS. (d) Patients were grouped by PFS >6 months and PFS <6 months for PPS. (e) Patients were grouped by the second phase of CHD >7 months and CHD ≤7 months for PFS. (f) Patients were grouped by CHD >7 months and CHD ≤7 months for PPS.

Median PFS, PPS, and OS were 6.9 months (95% CI, 5.0–8.6 months), 7.6 months (95% CI, 5.5–9.7 months), and 14.5 months (95% CI, 10.6–18.6 months), respectively (Figure 2(a)). One-year survival rate was 71.4% (95% CI, 53.6–89.3%). 24-week PFS rate was 60.7% (95% CI, 53.6–89.3%). 24-week PFS was 68.7% and 86.7% in the CHD group during and after chemotherapy and patients who received CHD >3 months, respectively.

Median PFS was 7 months in the CHD during and after chemotherapy group and 6.2 months in CHD after chemotherapy group (hazard ratio (HR), 0.88; 95% CI, 0.41–1.36; *P* = 0.57, Figure 2(b)). Median PFS was significantly longer in patients who received CHD >3 months compared with patients who received CHD ≤3 months in the first phase (8.7 months versus 4.5 months; HR, 0.52; 95% CI, 0.41–0.99; *P* = 0.0009, Figure 2(c)).

Median PPS was 9.6 months in PFS >6 months group and 7.6 months in PFS <6 months group (HR, 1.26; 95% CI, 0.83–1.70; *P* = 0.55, Figure 2(d)). Median PFS was not different between the two groups of patients who received CHD >7 months compared with patients who received CHD <7 months (7.2 months versus 6.1 months; HR, 1.18; 95% CI, 0.71–1.65; *P* = 0.97, Figure 2(e)). Median PPS was significantly longer in patients who received CHD >7 months compared with patients who received CHD ≤7 months (11.7 months versus 5.1 months; HR, 2.32; 95% CI, 1.90–2.74; *P* = 0.002, Figure 2(f)).

**Figure 3:**
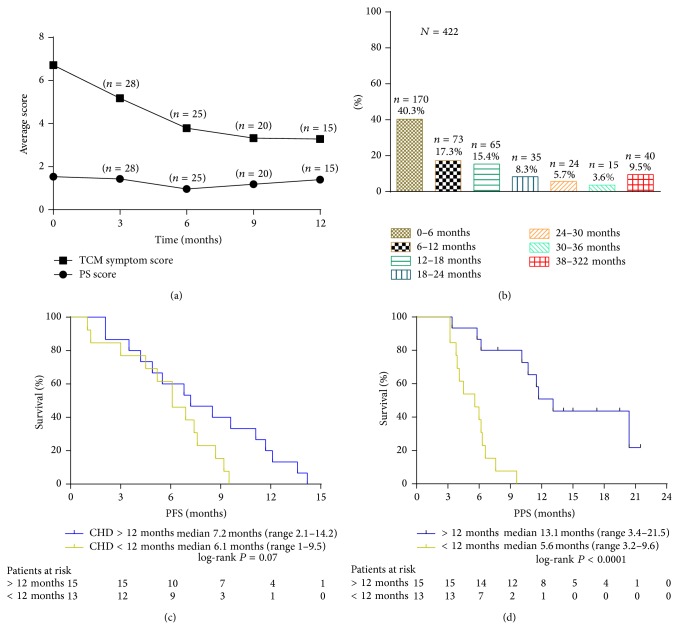
(a) Patients in a relatively stable state after 6 months of CHD treatment; (b) length of CHD treatment in 422 patients; ((c), (d)) patients were grouped by CHD >12 months and CHD <12 months for PFS and PPS, respectively.

Median PFS was not different between the two groups of patients who received CHD >12 months compared with patients who received CHD <12 months (7.2 months versus 6.1 months; HR, 1.18; 95% CI, 0.70–1.66; *P* = 0.07, Figure 3(c)). Median PPS was significantly longer in patients who received CHD >12 months compared with patients who received CHD <12 months (13.1 months versus 5.6 months; HR, 2.34; 95% CI, 1.91–2.77; *P* < 0.0001, Figure 3(d)).

### 3.5. Function Assessment

Function assessment included TCM syndromes and PS. The PS was evaluated by the doctors, and symptom measurements were made by the patients themselves and the doctors. In the first phase of follow-up 28 patients were included, and the assessment point-in-time was at the first visit and at the time when adjusting the prescription. In the second phase of follow-up one patient stopped taking CHD, and five patients died. The assessment point-in-time was after the disease progression and at the time when adjusting the prescription. In the first stage, the mean baseline PS score was 1.54, and the mean observed was 1.36, a mean drop of 0.18 points (95% CI, −0.16 to 0.53; *P* = 0.29; functional status improved). The mean base line TCM syndrome score was 6.71, and the mean observed was 5.18, a drop of 1.53 points (95% CI, 0.70–2.37; *P* = 0.001; TCM syndrome improved).  Table 5 shows that TCM worked in different stages for improving symptoms and quality of life, as well as stabilizing PS. During the clinical trial, patient's basic living state such as diet, sleep, urine, stool, and weight was stable.

### 3.6. Adverse Events

Bone marrow suppression was higher during chemotherapy, as were leukocytes (chemotherapy 75% versus CHD + chemotherapy 44%) and anemia (chemotherapy 50% versus CHD + chemotherapy 19%). The most obvious difference was fatigue in 91% of chemotherapy patients versus 25% of CHD + chemotherapy patients. Six (38%) patients had a grade 3/4 in the group of CHD + chemotherapy, compared with seven (58%) patients in the chemotherapy group (Table 6). Therefore, CHD improved fatigue and protected bone marrow. We found that the adverse reactions of CHD such as occasional diarrhea and vomiting could be improved by withdrawing or changing the prescription.

## 4. Discussion

To our knowledge, this is the first study on CHD syndrome differentiation therapy and its treatment length for ES-SCLC. Individualized treatment has been highlighted in the medical field and is a trend in comprehensive cancer treatment. TCM Zheng (syndrome) is a basic concept in TCM theory, and syndrome differentiation is the fundamental theory in TCM cancer treatment that can enable individualized treatment. The basic syndromes of lung cancer include Qi deficiency syndrome, Yin deficiency syndrome, and Phlegm syndrome. These syndromes connect with each other and can transfer to another under certain conditions. For example, Qi deficiency can develop into Yin deficiency and gradually form syndromes of Qi and Yin deficiency. Therefore, different fundamental prescriptions are often used together in clinical practice. According to the basic theory of TCM, we chose the herbs that tonify Qi to treat patients with Qi deficiency syndrome (Table 1(a)), the herbs that nourish Yin to treat patients with Yin deficiency syndrome (Table 1(b)), and the herbs that reduce phlegm to treat patients with Phlegm syndrome (Table 1(c)). Chinese herbal formulas are known to have an advantage with regard to bodily regulation [25, 26]. The aims of CHD treatment are to reduce toxic effects, prevent cancer recurrence and metastasis, slow tumor growth, and prolong tumor-bearing survival. Our results indicate that CHD may be a favorable treatment to improving prognosis in ES-SCLC.

In China, many patients take CHD in TCM clinics. It has been reported that 86.7% of cancer patients in China refer to CHM as one of their cancer treatments [27]. We hypothesize that long-term CHD treatment can change the microenvironment of the body, keep the body and tumor in balance, and delay the progression of disease and prolong the tumor-bearing survival time (including PFS and PPS). From May 7th through May 18th, 2012, 422 cancer patients visiting TCM oncology specialist clinics at Guang'anmen Hospital were interviewed about the duration of CHD cancer treatment. The results show that 179 (42.4%) patients took CHD for more than 1 year, 40 (9.5%) patients took CHD for more than 3 years, and the longest use of CHD treatment was 322 months (Figure 3(b)). The duration of CHD treatment for different cancer patients is a key problem, and further research is needed.

The current first-line treatment strategy for patients with extensive-stage disease is a combination of platinum and etoposide. A retrospective study in China showed that the median OS of ES-SCLC patients undergoing chemotherapy was 15.1 months, and the median PFS was 7.5 months [28]. A report showed similar results to ours with a median OS of 14.5 months and median PFS of 6.9 months, but the study did not mention whether these patients had taken CHD or not. Another study on Chinese patent medicine (CPM) as sustaining treatment for ES-SCLC patients showed that the median OS was 11.1 months [22]. A recent study indicated that, in a palliative setting (median OS in 304 patients, 9.4 months), therapeutic progress might not be obtained [29].

The median PFS of our study was 6.9 months, and the 24-week PFS was 60.7% (95% CI, 53.6–89.3%), compared with a history of 41% (95% CI, 18–65%) [10]. This indicates that CHD could prolong PFS when first-line therapy was effective and further increase patient sensitivity to second-line chemotherapy. SCLC tends to develop resistance, and second-line chemotherapy is based on the efficacy of the first-line chemotherapy [30]. In the first-line treatment, patients with progression within 3 months are considered to be “refractory.” However, patients who respond to first-line chemotherapy and then relapse after a treatment-free interval for more than 3 months are defined as “sensitive.” These patients are more likely to respond to second-line chemotherapy and may achieve a good prognosis with PFS >6 months [30, 31]. Currently, standard second-line chemotherapy for SCLC is not yet available. Topotecan as second-line chemotherapy was first recommended for patients who progress within 3–6 months and are sensitive to prior chemotherapy [32, 33].

Our findings show that there was no significant difference in PFS whether patients took CHD during chemotherapy or not, but PFS was closely related to the duration of CHD treatment. Median PFS was 8.7 months in the group taking CHD for more than 3 months, which is significantly longer than that in the group taking CHD for less than 3 months (*P* < 0.01). Our study suggests that using CHD for more than 3 months could prolong PFS, which may eventually increase the sensitivity to second-line chemotherapy. There was no remarkable difference in PFS between the group that took CHD during and after chemotherapy and the group that took CHD after chemotherapy (*P* = 0.57). However, the former had a lower rate of grade 3/4 (38% versus 58%), and the incidence rate of grade 1/2 was obviously lower than that in the group taking chemotherapy alone. Therefore, a combination of chemotherapy and CHD could reduce the toxicity of chemotherapy.

Our study indicates that CHD could prolong PPS to a large extent, which is related to the duration of CHD treatment. Median PPS was 7.6 months, which is longer than a reported second-line treatment for patients with relapsed or progressing disease with a median survival after relapse of 4-5 months [5]. To analyze PPS between patients taking CHD >7 months and ≤7 months, the possible effect of PFS on PPS was excluded. The results showed that the median PFS of the two groups were similar (7.2 months versus 6.1 months). In the group taking CHD >7 months, the number of patients with PFS >6 months was 8 (62%). In the group taking CHD ≤7 months, the number of patients with PFS >6 months was 9 (64%), which is not significantly different. However, a significant difference was found in PPS between the two groups with 11.7 months for the CHD group >7 months and 5.1 months for the CHD group ≤7 months (*P* = 0.002). This may suggest that longer duration for CHD treatment could prolong PPS. The first-line response was different between group taking CHD >7 months and ≤7 months. In further analysis 4 (14%) achieved CR, 7 (54%) achieved PR, and 2 (15%) had SD in the CHD >7 months group, while 7 (50%) patients achieved PR, and 7 (50%) achieved SD in the CHD ≤7 months group.

**Figure 4:**
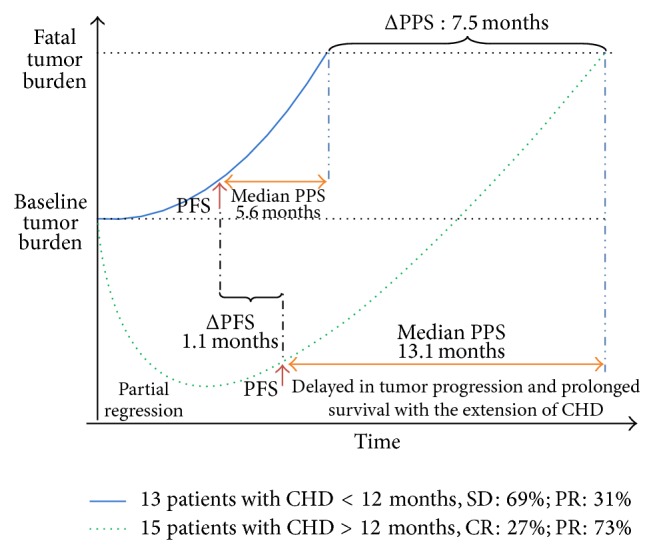
Patients received PPS longer than PFS with long-term CHD treatment. The extension of PPS was beneficial from early tumor shrinkage and deepness of response, providing the chance to use extended CHD treatment.

Our study suggests that the extension of PPS benefits from deepness of response (DpR) and long-term treatment with CHD. To further verify the results above, we analyzed the effect of total treatment time of CHD on PFS and PPS. We divided the patients into two groups according to their total treatment time with CHD, which were >12 months and <12 months. The results indicated that the median PFS of the two groups were similar (7.2 versus 6.1 months, Figure 3(c)). In the total treatment time >12 months group, the number of patients with PFS >6 months was 10 (60%). In the total treatment time <12 months group, the number of patients with PFS >6 months was 8 (62%), which was not obviously different. A significant difference was found in PPS between the two groups with 13.1 months and 5.6 months, respectively (Figure 3(d)). We also divided patients into two groups of PFS ≥6 months and PFS <6 months, and no difference was observed between the two groups in PPS. Thus, PFS may not be the most important factor to affect PPS of patients with ES-SCLC. Another cause for this significant difference may be that more PR and CR patients were included in the CHD >12 months group. It can be inferred that the difference in sensitivity of second-line chemotherapy between the two groups may also exist. In 2013, the ESMO 15th World Congress on Gastrointestinal Cancer proposed that a smaller tumor burden was associated with longer OS in metastatic colorectal cancer. Early tumor shrinkage (ETS) and DpR have significant effects on PFS, but less of an effect on PPS [34], and may eventually prolong OS. ESMO consensus recommends an “initial choice” of strong treatment to reduce tumor burden to the minimum. It is also noted that the efficacy of first-line treatment is the most important factor in determining survival time [34]. In our study, DpR may provide the opportunity for long-term CHD treatment and thus slow the progression of ES-SCLC. In this study population, patients with disease progression can still have a relatively long survival time, which may be the result of long-term use of TCM after first-line chemotherapy. TCM may prolong PFS and PPS of ES-SCLC patients by modulating residual tumors and reducing a tumor's invasive and metastatic potential (Figure 4).

The advantage of CHD is to relieve patient's current symptoms and improve quality of life. The effectiveness of CHD treatment is mostly determined by the improvement of PS and symptoms. Our study found that CHD can improve symptoms and PS to different degrees in the first and second stages of follow-up. TCM symptoms score and PS score were stable at 6 months (Figure 3(a)). At the same time, physicians should keep patients' diet, sleep, excrement, urine, and body weight stable to ensure the basic health of patients.

Both TCM and Western medicine have their advantages and disadvantages. The features of TCM are treatment based on symptom pattern differentiation and integrity of body functions. TCM focuses on macroscopic and external phenomenon, such as external clinical manifestations and adjustment for integrity of the human body internal environment [19]. However, western medicine focuses on microscopic and inner mechanisms, such as shrinkage of tumor size. Recent study indicates that the tumor microenvironment varies under different TCM syndromes. TCM could prevent tumor recurrence and metastasis and regulate the tumor microenvironment based on treatment response to herbal medicine. CHD may be a promising treatment for ES-SCLC by promoting recovery after surgery, reducing toxicity of chemotherapy, and improving the microenvironment of the body to prevent recurrence and metastasis. However, elucidating the mechanism of CHD is difficult, because CHD is a crude extract and complex composition that affects multiple targets.

Our study suggested that first-line chemotherapy is an effective time for CHD intervention. CHD could prolong PFS and PPS of ES-SCLC patients when chemotherapy was effective and is closely related to the duration of CHD treatment. Therefore, we suggest patients take CHD as early as possible to lengthen treatment time. The suggested duration of taking CHD is at least 3 months before disease progression and the total time of CHD treatment is not less than 12 months. This study may provide a basis for further TCM clinical studies based on syndrome differentiation.

## Figures and Tables

**Table:** (a) Qi deficiency syndrome.

Chinese name	Pharmaceutical name	*g*/*d*
Huang Qi	*RadixAstragali *	45
Tai Zi Shen	*RadixPseudostellariae *	15
Bai Zhu	*Atractylodes *	15
Fu Ling	*Wolfiporiaextensa *	20
Chen Pi	Pericarpium Citri Reticulatae	6

**Table:** (b) Yin deficiency syndrome.

Chinese name	Pharmaceutical name	*g*/*d*
Sha Shen	*Adenophoraelata *	30
Mai Dong	*Radix* Ophiopogonis	12
Sang Ye	Mulberry leaf	12
Xuan Shen	*Scrophularianingpoensis *	12
Shi Gao	Gypsum	45

**Table:** (c) Phlegm syndrome.

Chinese name	Pharmaceutical name	*g*/*d*
Gua Lou	Fructus Trichosanthis	20
Xie Bai	*Alliummacrostemon *	15
Xing Ren	Almond	10
Jie Geng	*Platycodongrandiflorum *	30
Ban Xia	*Pinelliaternata *	10

**Table:** (d) Anticancer (added 1-2 kinds to prescription).

Chinese name	Pharmaceutical name	*g*/*d*
Ban Zhi Lian	Barbed skullcap herb	30
Long Kui	Nightshade	15
Bai Ying	Bittersweet herb	20
Shi Jian Chuan	Chinese sage herb	15
Bai Hua She She Cao	Spreading hedyotis herb	30

Decocting method: soak the herbs in water for 30 min with water level 1 cm above the herbs. First, boil with strong heat, then with gentle heat for about 20–40 minutes. Then, decant the decoction, repeat the above course, combine the decoction, and concentrate to 300 mL.

Dosage and administration: one set of herbs per day, 150 mL each time, twice a day, one hour after breakfast and supper.

**Table 2:** ES-SCLC patient characteristics.

Characteristics	Time of CHD treatment (months)					
	First phase		Second phase		Total time	
	>3 (*n* = 15)	≤3 (*n* = 13)	>7 (*n* = 13)	≤7 (*n* = 14)	>12 (*n* = 15)	<12 (*n* = 13)
Median age (range)	60 (42–75)	62 (47–74)	61 (55–70)	60 (42–74)	61 (47–70)	60 (42–74)
Sex, (%)						
Men	11 (73)	11 (85)	10 (77)	11 (79)	11 (73)	11 (85)
Women	4 (27)	2 (15)	3 (23)	3 (21)	4 (27)	2 (15)
Smoking status index, (%)						
<400	3 (20)	3 (23)	2 (15)	4 (29)	4 (27)	2 (15)
≥400	9 (60)	8 (62)	9 (69)	7 (50)	9 (60)	8 (62)
No history of smoking	3 (20)	2 (15)	2 (15)	3 (21)	2 (13)	3 (23)
PS, (%)						
1	7 (47)	6 (46)	9 (69)	7 (50)	8 (53)	6 (46)
2	8 (53)	7 (54)	4 (31)	7 (50)	7 (47)	7 (54)
Site of metastasis, (%)						
Lung	6 (40)	9 (69)	7 (54)	8 (57)	7 (54)	8 (61)
Distant lymph nodes	15 (100)	13 (100)	13 (100)	14 (100)	15 (100)	13 (100)
Bone	2 (13)	—	1 (8)	1 (7)	1 (7)	1 (8)
Peritoneum	1 (7)	1 (8)	1 (8)	1 (7)	2 (13)	—
Pleura	1 (7)	—	1 (8)	—	1 (7)	—
Hydrothorax	1 (7)	—	1 (8)	—	1 (7)	—
Concomitant diseases, (%)						
Diabetes	5 (33)	3 (23)	4 (31)	4 (29)	8 (53)	3 (23)
Hypertension	1 (7)	3 (23)	3 (23)	1 (7)	2 (13)	2 (15)
Heart disease	1 (7)	1 (8)	2 (15)	—	2 (13)	—
Effectiveness of chemotherapy response, (%)						
Complete response	2 (13)	2 (14)	4 (31)	—	4 (27)	—
Partial response	7 (47)	8 (62)	7 (54)	7 (50)	11 (73)	4 (31)
Stable disease	4 (27)	5 (38)	2 (15)	7 (50)	—	9 (69)
Radiotherapy, (%)	7 (47)	8 (62)	6 (46)	9 (64)	7 (47)	8 (61)

**Table 3:** TCM syndrome response of one month after CHD treatment.

The main symptoms	Cases pretreatment (%)	Alleviated cases	Response rate (%)
Fatigue	21 (75)	17	80.9
Cough	18 (64)	15	83.3
Shortness of breath	17 (61)	10	58.8
Expectoration	16 (57)	14	87.5
Fever	6 (36)	4	66.7
Chest pain	5 (18)	1	20
Poor appetite	17 (61)	16	94.1
Insomnia	10 (36)	8	80
Constipation	17 (61)	15	51.9

**Table 4:** CHD treatment duration in each phase.

Time of CHD treatment	Median months (range)	Number of patients (%)
Taking CHD during and after chemotherapy	4.7 (2.6–11.1)	16 (57)
Taking CHD after chemotherapy	2.4 (1–10.7)	12 (43)
Total time	12.2 (3.2–27)	28
>12 months	17.2 (12.4–27)	15 (54)
<12 months	5.3 (3.2–10.5)	13 (46)
First phase	3.4 (1–11.1)	28
>3 months	6.7 (3.4–11.1)	15 (54)
≤3 months	1.8 (1–3)	13 (46)
Second phase	7.0 (1–22.5)	27
>7 months	12.7 (7.3–16.7)	13 (48)
≤7 months	2.6 (1–7.0)	14 (52)

**Table 5:** Function assessment in different phases.

Analysis	Baseline (I)		Observed (II)		Differences (I-II)		
	Mean	95% CI	Mean	95% CI	Mean	95% CI	*P*
PS							
First stage (*n* = 28)	1.54	1.34 to 1.73	1.36	1.07 to 1.64	0.18	−0.16to 0.53	0.29
Second stage (*n* = 23)	1.18	0.90 to 1.46	1.39	1.17 to 1.61	−0.21	−0.55 to 0.13	0.21
TCM syndromes							
First stage (*n* = 28)	6.71	5.36 to 8.14	5.18	4.36 to 6.11	1.53	0.70 to 2.37	0.001
Second stage (*n* = 22)	3.79	2.85 to 4.85	3.29	2.79 to 3.82	0.57	−0.45 to 1.45	0.29

**Table 6:** Changes in acute and subacute toxicity in the two groups after treatment.

Event, *n* (%)	CHD+ chemotherapy (*n* = 16)			Chemotherapy (*n* = 12)		
	Grade 1/2	Grade 3/4	Total (%)	Grade 1/2	Grade 3/4	Total (%)
Leukopenia	5 (31)	2 (13)	7 (44)	7 (58)	2 (17)	9 (75)
Anemia	3 (19)	—	3 (19)	4 (33)	2 (17)	6 (50)
Thrombocytopenia	2 (13)	1 (6)	3 (19)	3 (25)	—	3 (25)
Neutropenia	3 (19)	—	3 (19)	5 (42)	1 (8)	6 (50)
Fatigue	4 (25)	—	4 (25)	10 (83)	1 (8)	11 (91)
Nausea and vomiting	1 (6)	2 (13)	3 (19)	3 (25)	1 (8)	4 (33)
Diarrhea	3 (19)	1 (6)	4 (25)	1 (8)	—	1 (8)
Proteinuria	—	—	—	1 (8)	—	1 (8)

## Abbreviations

TCM: Traditional Chinese medicine
CHD: Chinese herbal decoction
CHM: Chinese herbal medicine
CPM: Chinese patent medicine
ES-SCLC: Extensive-stage small-cell lung cancer
OS: Overall survival
PFS: Progression-free survival
PPS: Postprogression survival
PS: World Health Organization performance status.
